# Self-injury functions mediate the association between anxiety and self-injury frequency among depressed Chinese adolescents: sex differences

**DOI:** 10.3389/fpsyt.2024.1378492

**Published:** 2024-05-23

**Authors:** Yunhan Zhao, Xudong Zhao, Yongjie Zhou, Liang Liu

**Affiliations:** ^1^ Clinical Research Center for Mental Disorders, Shanghai Pudong New Area Mental Health Center, School of Medicine, Tongji University, Shanghai, China; ^2^ Clinical Research Center for Mental Disorders, Chinese-German Institute of Mental Health, Shanghai Pudong New Area Mental Health Center, School of Medicine, Tongji University, Shanghai, China; ^3^ Shenzhen Mental Health Center, Shenzhen Kangning Hospital, Shenzhen, Guangdong, China

**Keywords:** anxiety, self-injury frequency, self-injury functions, depressed Chinese adolescents, mediation

## Abstract

**Objective:**

Non-suicidal self-injury (NSSI) has become a common clinical problem that severely threatens the mental and physical health of Chinese adolescents. This study explores the mediation effects of NSSI functions on the relationship between anxiety and NSSI frequency among depressed Chinese adolescents as well as the sex differences in the mediating effects.

**Methods:**

In this study, a cross-sectional survey method was used to obtain data of 1773 adolescent patients with major depressive disorders from over 20 specialized psychiatric hospitals across multiple provinces in China. A self-designed questionnaire for demographic information, the Chinese version of Functional Assessment of Self- Mutilation (C-FASM), and the 7-item Generalized Anxiety Disorder Scale (GAD-7) were employed to investigate demographic data, NSSI frequency, NSSI functions, and anxiety and to analyze the mediating effects of NSSI functions on the association between anxiety and NSSI frequency among adolescents of different sexes.

**Results:**

A total of 316 male patients and 1457 female patients were investigated. Female patients had a higher NSSI frequency (Z=3.195, P=0.001) and higher anxiety scores than did male patients (Z=2.714, P=0.007). Anxiety had a stronger positive predictive effect on the NSSI frequency in females (OR = 1.090) than in males (OR = 1.064). For male patients, the emotion regulation function in NSSI motivation played a full mediating role in the association between anxiety and NSSI frequency. For female patients, the emotion regulation and social avoidance functions in NSSI functions played a partial mediating role between anxiety and NSSI frequency.

**Conclusions:**

There are sex differences in the mediating role of NSSI functions of depressed adolescents in the association between anxiety and NSSI frequency. When experiencing anxiety, both males and females may engage in NSSI behaviors as a means to regulate their emotions. For females, anxiety can directly predict NSSI frequency, and they may attempt NSSI to achieve the purpose of rejecting others. In the face of anxiety among depressed adolescents of different sexes, developing different emotional regulation methods and behavioral regulation strategies may be critical in preventing their NSSI behaviors.

## 1 Introduction

Non-suicidal self-injury (NSSI) refers to a series of socially unacceptable behaviors in which an individual intentionally harms his or her own body without suicidal intent (1). Although NSSI behaviors can occur at any stage of an individual’s life, the incidence is the highest in adolescence (2, 3), constituting an independent risk factor for suicide and severely affecting the physical and mental health of adolescents (4). In recent years, the incidence of NSSI behaviors has continued to increase worldwide. A study in 41 countries found that young females are more prone than males to NSSI, with an average age of onset at 13 years. Among children and adolescents with mental disorders, the incidence of NSSI is about 50% (5), which is much higher than the NSSI prevalence of 17% observed in surveys of adolescent community samples (6).

### 1.1 Depression, anxiety, and NSSI

NSSI is associated with various mental disorders. A meta-analysis showed that individuals with mood disorders (anxiety, depression, and bipolar disorder) have a higher risk of exhibiting NSSI behaviors than did the normal group (7). Therefore, it is critical to study the interaction mechanisms between the emotions and NSSI behavior of adolescents with mood disorders. For example, numerous studies suggest that female sex, depression, and anxiety are risk factors for predicting NSSI behavior (8–11). Fox et al. (12) conducted a meta-analysis of NSSI risk factors and showed that depressive disorder is a risk factor for NSSI. Depression and NSSI often co-occur, with the presence of depression suggesting a significant increase in the incidence of NSSI behavior (13, 14). Clinically, the comorbidity rate of depression and anxiety among adolescents is as high as 15%-75% (15). Previous reports also showed that patients with anxious depression had more frequent episodes of major depression and a higher risk of suicidal ideation and previous suicide attempts than those with non-anxious depression (16). However, how anxiety affects adolescents’ NSSI behavior has not been fully studied.

### 1.2 NSSI functions and NSSI

NSSI functions refer to the motives or reinforcers of NSSI behavior (17). There have been many theoretical models proposed on the functions and purposes of NSSI behavior, including physiological models such as the homeostasis model proposed by Stanley et al. (18), psychological and social factor models such as the four-function model proposed by Nock and Prinstein (19), the developmental psychopathology framework proposed by Yates (20) the experiential avoidance model proposed by Chapman et al. (21), and the integrated motivational-volitional model proposed by O’Connor et al. (22). In their study of NSSI behavior in Chinese adolescents patients, Chinese researchers Qu et al. (23) proposed a three-factor model of NSSI functions that included emotion regulation, attention seeking, and social avoidance. On this basis, the Chinese version of Functional Assessment of Self-Mutilation (C-FASM) was formulated. The C-FASM has good content, structural validity, and reliability. The instrument can be helpful to Chinese adolescents as a comprehensive measure of NSSI behaviors. This model is applicable to both clinical and non-clinical populations within the Chinese cultural context. *Emotion regulation* refers to the behavior that patients exhibit to reduce or terminate unwanted negative emotional experiences (including anxiety, anger, and depression) through NSSI, so as to re-establish a sense of control. A number of studies have shown a significant positive correlation between negative cognitive emotion regulation and NSSI behaviors (24). *Attention seeking* means that adolescents engage in NSSI behaviors to seek social support and to gain attention and help from others. A relevant study (25) found that NSSI behaviors are closely related to positive social reinforcement and social influence. Among adolescents, the NSSI behavior of males, compared with females, is more often motivated by attention seeking and social influence. *Social avoidance* refers to adolescents’ avoidance of social needs, emphasizing that individuals use NSSI as a strategy to avoid adversity and social demands. Research on NSSI functions considers the repeated occurrence of NSSI as an outcome of *operant conditioning* (26): the specific situations before the behavior and behavioral outcomes exert (positive/negative/self) reinforcement on the individual. Once the individual encounters similar situations in the future, the same behavior will occur again. Therefore, if NSSI functions can be identified and the generation of behavioral outcomes associated with these functions can be controlled, an individual’s NSSI behavior will terminate.

### 1.3 Sex and NSSI

Sex is a key factor affecting all aspects of adolescents’ physical and mental health. Universal sex differences exist in pathological conditions including anxiety and depression, with females being more than twice as likely to develop the disease as males (27), which may be related to differences in various aspects such as brain structure and function, sex hormones, and social expectations (28). Moreover, there are distinct sex differences in NSSI behaviors between males and females. Researchers have confirmed (29, 30) that the incidence of NSSI is higher in females than in males, indicating that females may be more inclined than males to resort to NSSI behaviors to alleviate overwhelming distress. A study has shown (31) that adolescent females are more emotionally sensitive than are adolescent males, suggesting that they are more susceptible to perceiving and absorbing negative emotions. Therefore, this study considers the impact of anxiety in adolescent patients of different sexes on NSSI behaviors.

**Figure 1:**
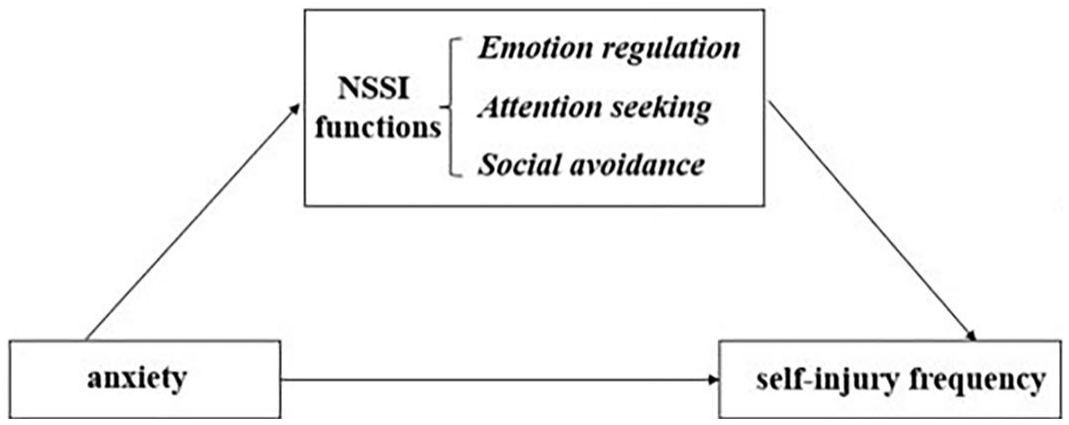
The mediating effects of NSSI functions.

Although previous studies have confirmed that anxiety, NSSI functions, and sex are all associated with NSSI, the interaction mechanisms between anxiety and NSSI functions in the process of triggering NSSI and whether there are sex differences in these mechanisms remain unclear. Additionally, previous research findings have mostly focused on non-clinical samples, mainly through large-scale epidemiological surveys of middle school students; there are relatively few studies that include clinical samples. Therefore, in the present study, a sample of Chinese adolescents with depressive disorders is used to investigate the mediating effects of NSSI functions in the process by which anxiety triggers NSSI behaviors and to examine the sex difference in these effects (see **Figure 1** for the hypotheses).

## 2 Methods

### 2.1 Participants

In this study, three-stage sampling was used to select the research participants. First, nine provinces in China were selected based on their economic conditions [three were good, three were moderate, and three were poor); second, 11 specialized psychiatric hospitals and the psychiatric departments of 9 general hospitals (20 institutions in total)] in these nine provinces were selected (All these institutions were willing to participate in the survey and had professional research team to carried out the study); and finally, outpatient and inpatient adolescents with depressive disorder were recruited by convenience sampling. From the beginning of recruitment in December 2020 to its completion in May 2022, the data of a total of 1773 adolescent patients with depressive disorders were collected. The inclusion criteria were as follows: (1) meeting the diagnostic criteria for major depressive disorders in the Fifth Edition of the Diagnostic and Statistical Manual of Mental Disorders (DSM-5); (2) aged 12-18 years; (3) at least one instance of NSSI behavior in the past; (4) a minimum of six years of education, so they could understand the questions in the questionnaire and complete the survey independently; (5) patients and their families consenting to participate in this project and signing an informed consent form. The exclusion criteria were as follows: (1) patients with severe physical illnesses, infectious diseases, and immune system disorders; (2) patients with a history of traumatic brain injury, epilepsy, or other known severe neurological diseases or organic brain diseases; and (3) patients with a history of severe mental disorders such as schizophrenia or intellectual developmental disorder. This study was approved by the Ethics Committee of Tongji University as well as the Shanghai Pudong New Area Mental Health Center (No.PKJ2023-Y21).

### 2.2 Measures

#### 2.2.1 Sociodemographic data and relevant clinical information

A demographic questionnaire was developed to collect the following information: sex, age, date of birth, years of education, place of residence, family income, parental education level, family history of mental disorders, presence of physical illnesses, previous diagnosis of mental disorders (first episode or not), and the type of currently diagnosed disease (to be completed after an evaluation by a doctor).

#### 2.2.2 Functional assessment of self-mutilation (Chinese version), C-FASM

The FASM scale was developed by Lloyd et al. (32) in 1997 to assess adolescent NSSI behaviors and NSSI functions. Considering the influence of time and cultural differences, Qu et al. (23) made revisions (C-FASM) to make it more suitable for the evaluation of Chinese patients. This scale evaluates the frequency and functions of NSSI behaviors of respondents over the past 12 months. The C-FASM scale consists of a 15-item NSSI functional checklist as well as other NSSI characteristics such as age at onset of NSSI and NSSI frequency. The Chinese version of the FASM has been demonstrated to have good content validity as well as good construct validity and reliability, making it a comprehensive measurement tool for NSSI behaviors (23).

In the C-FASM, NSSI functions are divided into three categories: emotion regulation (5 items, eg., ‘*to relieve feeling numb or empty*’), attention seeking (6 items, eg., ‘*to get attention’*), and social avoidance (4 items, eg., ‘*to avoid school, work, or other activities*’). In present study, Cronbach’ α coefficients for emotion regulation, attention seeking, and social avoidance are 0.72, 0.87, and 0.72, respectively.

C-FASM also describes 10 common ways (eg.,. ‘*cut or carved on your skin, hit yourself on purpose*, and *picked at a wound*’) that adolescents engage in self-injury. We asked participants how often they self-harmed in these ways in past 12 months. Then, we summarized the frequency of NSSI in 10 ways into the overall frequency of NSSI. The overall frequency of NSSI ranged from 0 to 1519 (*M* = 198.50, *SD* = 287.60). The average score of overall frequency of NSSI for males was 177.70 (*SD* = 290.26) and for females was 203.02 (*SD* = 286.92). According to previous studies, we divided The overall frequency of NSSI into 5 levels: 0 times (0 = never), 1-13 times (1= rarely), 14-38 times (2= occasionally), 39-106 times (3 = often), and >106 times (4 = frequently). After converting to 5 levels, the average score of *NSSI frequency* was 2.75 (*SD* = 1.21).

#### 2.2.3 The 7-item generalized anxiety disorder scale

The GAD-7 is a concise anxiety self-assessment scale developed by Spitzer et al. (33) based on the diagnostic criteria of generalized anxiety disorder in the Fourth Edition of the Diagnostic and Statistical Manual of Mental Disorders (DSM-IV) in the United States. Studies have shown that the GAD-7 has high reliability and validity for anxiety screening (34, 35). The GAD-7 consists of seven items, each of which is scored on a 4-point scale from 0 to 3, with the total score serving as the primary indicator. Higher scores indicate greater severity of anxiety. In this study, Cronbach’ α coefficients for anxiety is 0.92.

### 2.3 Statistical analysis

SPSS27.0 was used for statistical analyses. Count data are expressed as the number of cases and percentage (%). The χ^2^ test was used for between-group comparisons of categorical variables, and t-test was performed to compare continuous variables. Regression analysis was used to evaluate the effect of anxiety on NSSI functions and frequency, and the nonparametric Spearman rank test was conducted to evaluate the correlation between NSSI frequency and functions. Model 4 in the SPSS macro developed by Hayes was used to test the mediating effects of NSSI motivation on the association between anxiety and NSSI frequency for the male group and for the female group after controlling for confounding factors such as age and previously diagnosed mental disorders (36).

## 3 Results

### 3.1 Demographic characteristics of the study participants

In this study, the data of a total of 1773 adolescents with depressive episodes and NSSI behaviors, including 316 males (17.82%) and 1457 females (82.18%), were analyzed. The age of male adolescents (15.29 ± 1.519 years) was higher than that of females (14.76 ± 1.652 years); the difference was statistically significant (F=5.245, P<0.001). There was also a statistically significant difference in whether they had been previously diagnosed with mental disorders (F=6.960, P=0.008). However, there were no statistically significant differences between the two groups in terms of place of residence, family income, parental education level, family history of mental disorders, presence of physical illnesses, and types of mood disorders.

### 3.2 Sex differences in NSSI frequency, NSSI functions, and anxiety symptoms

The frequency of NSSI behaviors in patients was evaluated using the FASM. The patients were divided into five levels based on the self-reported number of instances of NSSI behaviors in the past 12 months: 0 times (never), 1-13 times (rarely), 14-38 times (occasionally), 39-106 times (often), and >106 times (frequently). Regarding NSSI behavior frequency levels, there was a statistically significant difference between males and females (X^2 ^= 3.195, P=0.001, Cramer’s V = 0.115); the proportion of patients at the “often” and “frequently” levels was significantly higher for female patients than for male patients, and females had a higher NSSI frequency than did males (**Table 1**).

**Table 1:** Sex differences in NSSI frequency, NSSI functions, and anxiety scale scores.

Variable	Male (n = 316)	Female (n = 1457)	χ^2^ value/t value	P value	Cohen’s d/Cramer’s V
NSSI frequency [n (%)]					
Never	10 (3.2)	11 (0.8)	23.338^a^	0.001	0.115
Rarely	79 (25.0)	290 (19.9)			
Occasionally	70 (22.2)	247 (18.8)			
Often	47 (14.9)	295 (20.2)			
Frequently	110 (34.8)	587 (40.3)			
NSSI functions					
Emotion regulation	12.83 ± 4	14.13 ± 3.917	-5.336^b^	<0.001	-0.331
Attention seeking	10.74 ± 4.806	10.05 ± 4.665	2.380^b^	0.017	0.148
Social avoidance	7.38 ± 3.230	7.22 ± 3.116	0.803^b^	0.442	0.050
Anxiety scale score	12.04 ± 6.135	13.05 ± 5.991	2.724^b^	0.007	-0.169

a: x^2^ value; b: t value;

The analysis of the NSSI functions in the FASM revealed that males and females differed in emotion regulation (t=-5.336, P<0.001, Cohen’s d = -0.331) and attention seeking (t=2.380, P=0.017, Cohen’s d = 0.148) and that the NSSI functions of females were more oriented toward emotion regulation while those of males were more oriented to attention seeking (**Table 1**).

Regarding the GAD-7 results, there was a significant difference in anxiety scale scores between males and females (t=2.724, P=0.007, Cohen’s d = -0.169), with female patients having higher anxiety scores than male patients (**Table 1**).

### 3.3 Influence of anxiety on NSSI frequency: sex differences

The participants were divided into two groups by sex, each of which was subjected to ordinal regression. We constructed two ordinal regression models to explore the factors influencing the frequency of NSSI in male and female respectively. In all models, sex, age, family income, and previously diagnosed mental disorders were entered as predictors, NSSI frequency was entered as a dependent variable. The results of the parallel lines test for the male group was *χ* ^2 ^= 13.418, df = 12, *p* > 0.05, indicating the existence of the proportional advantage hypothesis. The analysis results (**Table 2**) showed that factors associated with NSSI frequency in males included anxiety and age (*p* < 0.05). The result of the parallel lines test for the female group was *χ* ^2 ^= 15.923, df = 12, *p* > 0.05, indicating the existence of the proportional advantage hypothesis. Factors associated with NSSI frequency in females included anxiety, age, and previously diagnosed mental disorders (*p* < 0.05). Anxiety had a stronger positive predictive effect on the increase in NSSI frequency for females (OR=1.090) than for males (OR=1.064).

**Table 2:** Ordinal regression analysis of factors influencing the NSSI frequency in patients by sex.

Sex	Variable	*B*	95% confidence interval for *B*		*SE*	*Wald* value	*P* value	*OR*
			Lower limit	Upper limit				
Male	Anxiety	0.062	0.028	0.095	0.017	13.102	0.000	1.064
	Age	-0.148	-0.282	-0.014	0.068	4.705	0.030	0.862
	Previously diagnosed mental disorders	0.250	-0.359	0.858	0.310	0.647	0.421	1.284
Female	Anxiety	0.086	0.070	0.103	0.008	106.005	0.000	1.090
	Age	-0.100	-0.158	-0.041	0.030	11.234	0.001	0.905
	Previously diagnosed mental disorders	0.252	0.007	0.496	0.125	4.070	0.044	1.287

### 3.4 Influence of anxiety on NSSI functions: sex differences

The participants were divided into two groups by sex, with 316 males and 1457 females, and regression analysis was performed separately for each group. The results of sex stratified analysis (**Table 3**) showed that the positive predictive effect of anxiety on the three NSSI functions was present in both the male and female groups (*ps <*0.001), with the positive predictive effect of anxiety on emotion regulation, attention seeking, and social avoidance being slightly higher in males than in females.

**Table 3:** Influence of anxiety on NSSI functions in patients by sex.

Variable	Emotion regulation		Attention seeking		Social avoidance	
	*β*	*t*	*β*	*t*	*β*	*t*
Male						
Independent variable						
Anxiety	0.423	8.213^***^	0.277	5.074^***^	0.332	6.179^***^
Control variables						
Age	-0.021	-0.405	-0.072	-1.317	-0.005	-0.100
Previously diagnosed mental disorders	0.025	0.490	0.030	0.548	0.033	0.607
*R* ^2^		0.187		0.088		0.119
Adjusted *R* ^2^		0.177		0.076		0.107
*F*		17.908^***^		7.490^***^		10.475^***^
Female						
Independent variable						
Anxiety	0.358	14.403^***^	0.163	6.230^***^	0.264	10.266^***^
Control variables						
Age	0.007	0.288	0.047	1.781	-0.030	-1.147
Previously diagnosed mental disorders	0.041	1.679	0.034	1.310	0.011	0.422
*R* ^2^		0.133		0.035		0.074
Adjusted *R* ^2^		0.131		0.032		0.071
*F*		55.864^***^		13.003^***^		28.984^***^

*** p < 0.001.

### 3.5 Correlations between NSSI frequency and functions: sex differences

The participants were divided into two groups by sex, with 316 males and 1457 females. Due to non-normal distributions of NSSI frequency and functions for each group, a nonparametric Spearman rank test was conducted separately for the two groups. Statistical analysis showed a significant correlation between frequency and the three functions of NSSI for both males and females (**Table 4**). Effect sizes were evaluated according to Cohen’s (37) benchmarks: correlations of *r* = .10 to .30 were considered small, .30 to .50 were considered medium, and over .50 were considered large. The result (**Table 4**) showed that emotion regulation had a moderate and significant correlation with NSSI frequency. Social avoidance and attention seeking both had a small and significant correlations with NSSI frequency.

**Table 4:** Correlation between NSSI frequency and functions by sex.

Variable	NSSI frequency [n]			
	Male (n = 316)		Female (n = 1457)	
	R	P value	R	P value
NSSI functions				
Emotion regulation	0.456	< 0.001	0.431	< 0.001
Attention seeking	0.189	< 0.001	0.108	< 0.001
Social avoidance	0.281	< 0.001	0.215	< 0.001

### 3.6 Mediation analysis

The participants were divided into two groups by sex. Using Model 4 in the SPSS macro developed by Hayes (36), the mediating effects of NSSI motivation on the relationship between anxiety and NSSI frequency was tested separately for the male and female groups while controlling for age and previously diagnosed mental disorders. The results for the male group (**Tables 5**, **6**) showed that anxiety had a significant predictive effect on NSSI frequency (*β* = 0.206, *t* = 3.727, *p* < 0.001). However, after including the mediator variables, the direct predictive effect of anxiety on NSSI frequency was not significant (*β* = 0.005, *t* = 0.094, *p* > 0.05). Anxiety had a significant positive predictive effect on the three types of NSSI functions (*ps* < 0.001), and the emotion regulation function in NSSI functions had a significant positive predictive effect on NSSI frequency (*β* = 0.415, *t* = 6.970, *p* < 0.001). In addition, both the upper and lower limits of the bootstrap 95% confidence intervals for the mediating effects of the emotion regulation functions excluded 0 (**Table 6**), indicating that emotion regulation in NSSI functions played a full mediating role in the relationship between anxiety and NSSI frequency and that anxiety predicted NSSI frequency through emotion regulation in NSSI functions. The mediating effect of emotion regulation (0.176) accounted for 82.243% of the total effect (0.214).

**Table 5:** Test of the mediation model of the three types of NSSI functions by sex.

Variable	NSSI frequency		Emotion regulation		Attention seeking		Social avoidance		NSSI frequency	
	*β*	*t*	*β*	*t*	*β*	*t*	*β*	*t*	*β*	*t*
Male										
Age	-0.117	-2.128^*^	-0.021	-0.405	-0.072	-1.317	-0.005	-0.1	-0.106	-2.094^*^
Previously diagnosed mental disorders	0.042	0.752	0.025	0.490	0.030	0.548	0.033	0.607	0.029	0.564
Anxiety	0.206	3.727^***^	0.423	8.213^***^	0.277	5.074^***^	0.332	6.179^***^	0.005	0.094
Emotion regulation									0.415	6.970^***^
Attention seeking									0.035	0.613
Social avoidance									0.046	0.759
* R*		0.257		0.433		0.296		0.345		0.476
* R* ^2^		0.066		0.187		0.088		0.119		0.227
* F*		5.497^***^		17.908^***^		7.490^***^		10.475^***^		12.918^***^
Female										
Age	-0.089	-3.484^**^	0.007	0.288	0.047	1.781	-0.03	-1.147	-0.087	-3.661^***^
Previously diagnosed mental disorders	0.045	1.802	0.041	1.679	0.034	1.31	0.011	0.422	0.031	1.336
Anxiety	0.272	10.700^***^	0.358	14.403^***^	0.163	6.230^***^	0.264	10.266^***^	0.131	5.126^***^
Emotion regulation									0.363	13.898^***^
Attention seeking									-0.050	-1.921
Social avoidance									0.073	2.681^**^
* R*		0.305		0.365		0.186		0.272		0.468
* R* ^2^		0.093		0.133		0.035		0.074		0.219
* F*		37.112^***^		55.864^***^		13.003^***^		28.984^***^		57.899^***^

*p < 0.05, **p < 0.01, ***p < 0.001.

**Table 6:** The total effect and direct effect of anxiety on NSSI frequency and the mediating effects of NSSI functions.

	Effect size	Bootstrap standard error	Bootstrap CI lower limit	Bootstrap CI upper limit	Relative effect size (%)
Male					
Total effect	0.214	0.057	0.101	0.327	
Direct effect	0.006	0.059	-0.111	0.122	2.804
Mediating effect of emotion regulation	0.176	0.033	0.115	0.245	82.243
Mediating effect of attention seeking	0.01	0.016	-0.022	0.041	4.673
Mediating effect of social avoidance	0.015	0.023	-0.028	0.062	7.009
Female					
Total effect	0.269	0.025	0.220	0.318	
Direct effect	0.130	0.025	0.080	0.179	48.327
Mediating effect of emotion regulation	0.130	0.013	0.105	0.156	48.327
Mediating effect of attention seeking	-0.008	0.004	-0.017	0.000	-2.974
Mediating effect of social avoidance	0.019	0.007	0.006	0.034	7.063

The results for the female group (**Tables 5**, **6**) showed that anxiety had a significant predictive effect on NSSI frequency (*β* = 0.272, *t* = 10.700, *p* < 0.001). Furthermore, after the mediator variable was included, the direct predictive effect of anxiety on NSSI frequency remained significant (*β* = 0.131, *t* = 5.126, *p* < 0.001). Anxiety had a significant positive predictive effect on all three types of NSSI functions (*ps* < 0.001), and emotion regulation (*β* = 0.363, *t* = 13.898, *p* < 0.001) and social avoidance (*β* = 0.073, *t* = 2.681, *p* < 0.01) in NSSI functions also had significant positive predictive effects on NSSI frequency. In addition, both the upper and lower limits of the bootstrap 95% confidence intervals for the direct effect of anxiety on NISS frequency and the mediating effects of emotion regulation and social avoidance excluded 0 (**Table 6**), indicating that anxiety could not only directly predict NSSI frequency but also predict NSSI frequency through the mediating effects of emotion regulation and social avoidance in NSSI functions. The direct effect (0.130), the mediating effect of emotion regulation (0.130), and the mediating effect of social avoidance (0.019) accounted for 48.327%, 48.327%, and 7.063% of the total effect (0.269), respectively.

## 4 Discussion

In this study, for the first time, the mediating effects of NSSI functions on the association between anxiety and NSSI behaviors in adolescents with depressive disorders, as well as the sex difference in these effects, were investigated. First, the frequency of NSSI behaviors was higher in females than in males, and higher proportions of females reported NSSI behaviors at “often” and “frequently” levels, findings that are consistent with the results of most previous studies. This could be because females, compared to males, employ different emotion regulation strategies when coping with their own stress and are more susceptible to emotional issues such as anxiety and depression, consequently resulting in more NSSI behaviors (29). This explanation was partially supported in the present study through the analysis of NSSI functions of different sexes: the functions of females were more oriented to emotion regulation while those of males were more oriented toward the attention seeking. There was no statistically significant difference between males and females in social avoidance, a finding that aligns with the result of a previous study (25).

Second, there are few studies on the relationship between anxiety and NSSI behaviors. The existing studies have shown a significant correlation between anxiety and NSSI behavior (3, 13, 38–40), with a higher level of anxiety leading to a higher probability of occurrence of NSSI behaviors. As a possible explanation, during the NSSI process, patients may adopt more dysfunctional ways to regulate their negative emotions and thus alleviate their anxiety; on the other hand, NSSI behaviors may exacerbate anxiety to some extent, leading to a vicious cycle that further intensifies negative emotions and NSSI behaviors (41, 42).

Moreover, the results of this study suggest that for female patients, anxiety can directly predict NSSI behaviors. This phenomenon may be attributed to differences in physiological mechanisms, hormone levels, and psychological development between sexes. Females enter adolescence earlier than do males and face more psychological issues and distress, thus experiencing more pronounced anxiety. Furthermore, there is an association between female NSSI behaviors and menarche, with NSSI behaviors possibly serving as a way for females to cope with adverse emotions related to menarche and menstrual anxiety (43).

Finally, the main finding of the present study is that *emotion regulation* works as a common mediator in the association between anxiety and NSSI frequency for both males and females; in females, *social avoidance* also play partial mediating roles in the relationship between anxiety and NSSI frequency. For both sexes, anxiety affected NSSI frequency through the *emotion regulation* function in NSSI functions, suggesting that depressed adolescents may engage in NSSI behaviors to regulate emotions when they experience anxiety. This may be related to a variety of factors from different ecological systems, such as sociocultural norms, school and educational environments, peer relationship and family interpersonal atmosphere (25, 44, 45).Traditional Chinese educational philosophy emphasizes the importance of children being responsible, respectful, mature, and disciplined. Moreover, it’s common for teenagers to face challenges with interpersonal relationships on-campus as well as their studies, while their home environment may lack openness and encourage emotional restraint (46).This may potentially cause the adolescents in this study to be more inclined to suppress their emotions and not express them directly when they experience anxiety from multi-ecological systems, thus causing them to lean toward NSSI as a way to cope with anxiety (47).

For female patients, the present study showed that *social avoidance* play partial mediating roles in the association between anxiety and NSSI behaviors, suggesting that it can also explain part of the mechanisms by which anxiety triggers NSSI behaviors in depressed females. These factors could also be influenced by societal norms, the educational atmosphere, and the family dynamics within multi-ecological systems. First, traditional Chinese culture requires females to be more submissive, causing them to experience anxiety when faced with unreasonable expectations from other people (48, 49). Second, Chinese society, along with its education system and many parents, places significant importance on the academic achievements of teenagers. Furthermore, in our research, we’ve observed that some parents in China tend to adopt a more authoritarian approach to parenting (47, 50). Hence, the female adolescents may not dare to refuse directly and can only adopt NSSI as an indirect way to reject and avoid demands from society, teachers, peers and families. However, our results demonstrated that the prediction effect of anxiety on NSSI behaviors still remains when the mediating effects of *emotion regulation* and *social avoidance* are counted. It might implied about the direct influence of female’s anxiety on their NSSI behaviors, as well as the existence of other potential mediators between female’s anxiety and NSSI behaviors. Hence, future research including more potential variables is strongly suggested to explore the complex mechanisms by which anxiety, NSSI functions and these variables triggers self-injuries behaviors in depressed adolescents.

This study also has the following limitations. First, this investigation was cross-sectional in design, and a longitudinal study is needed to confirm the causal relationship among anxiety, NSSI functions, and NSSI behaviors. Second, NSSI behaviors in adolescents with depressive disorders are closely related to biological, psychological, and social factors. In this study, biological and other sociological factors were considered to a lesser extent, and analyses were conducted only at the emotional level. In future research, other factors such as family factors, interpersonal relationships, and adverse life events should be further discussed and analyzed.

## 5 Conclusions

The results of this study show that *emotion regulation* plays an important mediating role in the association between anxiety and NSSI frequency among depressed adolescents. This suggests that in clinical interventions for depressed adolescents with anxiety, clinicians should consider discussing with patients more emotion regulation strategies to indirectly reduce the risk of NSSI (51). For depressed female adolescents who engage in NSSI, it may be beneficial to discuss with them how to better express their demands in interpersonal communication, as well as how to reject others’ unreasonable expectations and demands. This may reduce their risk of NSSI more effectively (52).

## Data availability statement

The raw data supporting the conclusions of this article will be made available by the authors, without undue reservation.

## Ethics statement

This study was approved by the Ethics Committee of Tongji University as well as the Shanghai Pudong New Area Mental Health Center (No.PKJ2023-Y21).

## Author contributions

YuZ: Data curation, Investigation, Writing – original draft. XZ: Project administration, Resources, Supervision, Writing – review & editing. YoZ: Formal analysis, Methodology, Resources, Writing – review & editing. LL: Conceptualization, Supervision, Validation, Writing – review & editing.

## References

[1] Kiekens G Claes L. Non-suicidal self-injury and eating disordered behaviors: an update on what we do and do not know. Curr Psychiatry Rep. (2020) 22:68. doi: 10.1007/s11920-020-01191-y 33037934 PMC7547297

[2] Bježančević M Hržić IG Dodig-Ćurković K. Self-injury in adolescents: a five-year study of characteristics and trends. Psychiatr Danub. (2019) 31:413–20. doi: 10.24869/psyd.2019.413 31698397

[3] Thippaiah SM Nanjappa MS Gude JG Voyiaziakis E Patwa S Birur B. Non-suicidal self-injury in developing countries: a review. Int J Soc Psychiatry. (2021) 67:472–82. doi: 10.1177/0020764020943627 32715834

[4] Brausch AM Muehlenkamp JJ. Perceived effectiveness of NSSI in achieving functions on severity and suicide risk. Psychiatry Res. (2018) 265:144–50. doi: 10.1016/j.psychres.2018.04.038 PMC5984167 29709788

[5] Gillies D Christou MA Dixon AC Featherston OJ Rapti I Garcia-Anguita A. Prevalence and characteristics of self-harm in adolescents: meta-analyses of community-based studies 1990-2015. J Am Acad Child Adolesc Psychiatry. (2018) 57:733–41. doi: 10.1016/j.jaac.2018.06.018 30274648

[6] Klimes-Dougan B Begnel E Almy B Thai M Schreiner MW Cullen KR. Hypothalamic-pituitary-adrenal axis dysregulation in depressed adolescents with non-suicidal self-injury. Psychoneuroendocrinology. (2019) 102:216–24. doi: 10.1016/j.psyneuen.2018.11.004 30590339

[7] Meszaros G Horvath LO Balazs J. Self-injury and externalizing pathology: a systematic literature review. BMC Psychiatry. (2017) 17:160. doi: 10.1186/s12888-017-1326-y 28468644 PMC5415783

[8] Bae SH Sung HJ. A study on the factors influencing the non-suicidal self-injury of middle school students: focusing on the severity of self-injury. Ment Health Soc Work. (2020) 48:122–48. doi: 10.24301/MHSW.2020.06.48.2.122

[9] Huang C Chen JH. Meta-analysis of the factor structures of the beck depression inventory-II. Assessment. (2015) 22:459–72. doi: 10.1177/1073191114548873 25172846

[10] Kang SG Lee YJ Kim SJ Lim W Lee HJ Park YM. Weekend catch-up sleep is independently associated with suicide attempts and self-injury in Korean adolescents. Compr Psychiatry. (2014) 55:319–25. doi: 10.1016/j.comppsych.2013.08.023 24267542

[11] Valencia-Agudo F Burcher GC Ezpeleta L Kramer T. Nonsuicidal self-injury in community adolescents: a systematic review of prospective predictors, mediators and moderators. J Adolesc. (2018) 65:25–38. doi: 10.1016/j.adolescence.2018.02.012 29522914

[12] Fox KR Franklin JC Ribeiro JD Kleiman EM Bentley KH Nock MK. Meta-analysis of risk factors for nonsuicidal self-injury. Clin Psychol Rev. (2015) 42:156–67. doi: 10.1016/j.cpr.2015.09.002 PMC4772426 26416295

[13] Zhang P Ouyang L Liang M Wu Y Bao C Yang K. A cross-sectional epidemiological study of non-suicidal self-injury prevalence in Chinese psychiatric patients. Nat Ment Health. (2023) 1:266–72. doi: 10.1038/s44220-023-00050-y

[14] Barrocas AL Giletta M Hankin BL Prinstein MJ Abela JR. Nonsuicidal self-injury in adolescence: longitudinal course, trajectories, and intrapersonal predictors. J Abnorm Child Psychol. (2015) 43:369–80. doi: 10.1007/s10802-014-9895-4 24965674

[15] Cummings CM Caporino NE Kendall PC. Comorbidity of anxiety and depression in children and adolescents: 20 years after. Psychol Bull. (2014) 140:816–45. doi: 10.1037/a0034733 PMC4006306 24219155

[16] Choi KW Kim YK Jeon HJ. Comorbid anxiety and depression: clinical and conceptual consideration and transdiagnostic treatment. Adv Exp Med Biol. (2020) 1191:219–35. doi: 10.1007/978-981-32-9705-0_14 32002932

[17] Klonsky ED Muehlenkamp JJ. Self-injury: a research review for the practitioner. J Clin Psychol. (2007) 63:1045–56. doi: 10.1002/jclp.20412 17932985

[18] Stanley B Sher L Wilson S Ekman R Huang YY Mann JJ. Non-suicidal self-injurious behavior, endogenous opioids and monoamine neurotransmitters. J Affect Disord. (2010) 124:134–40. doi: 10.1016/j.jad.2009.10.028 PMC2875354 19942295

[19] Nock MK Prinstein MJ. A functional approach to the assessment of self-mutilative behavior. J Consult Clin Psychol. (2004) 72:885–90. doi: 10.1037/0022-006X.72.5.885 15482046

[20] Yates TM. The developmental psychopathology of self-injurious behavior: compensatory regulation in posttraumatic adaptation. Clin Psychol Rev. (2004) 24:35–74. doi: 10.1016/j.cpr.2003.10.001 14992806

[21] Chapman AL Gratz KL Brown MZ. Solving the puzzle of deliberate self-harm: the experiential avoidance model. Behav Res Ther. (2006) 44:371–94. doi: 10.1016/j.brat.2005.03.005 16446150

[22] O'Connor RC Rasmussen S Hawton K. Distinguishing adolescents who think about self-harm from those who engage in self-harm. Br J Psychiatry. (2012) 200:330–5. doi: 10.1192/bjp.bp.111.097808 22403089

[23] Qu D Wang Y Zhang Z Meng L Zhu F Zheng T. Psychometric properties of the Chinese version of the functional assessment of self-mutilation (FASM) in Chinese clinical adolescents. Front Psychiatry. (2022) 12:755857. doi: 10.3389/fpsyt.2021.755857 35153848 PMC8826685

[24] Brereton A McGlinchey E. Self-harm, emotion regulation, and experiential avoidance: a systematic review. Arch Suicide Res. (2020) 24:1–24. doi: 10.1080/13811118.2018.1563575 30636566

[25] You J Lin MP Leung F. Functions of nonsuicidal self-injury among Chinese community adolescents. J Adolesc. (2013) 36:737–45. doi: 10.1016/j.adolescence.2013.05.007 23849668

[26] Carr EG Taylor JC Robinson S. The effects of severe behavior problems in children on the teaching behavior of adults. J Appl Behav Anal. (1991) 24:523–35. doi: 10.1901/jaba.1991.24-523 PMC1279602 1752841

[27] McHenry J Carrier N Hull E Kabbaj M. Sex differences in anxiety and depression: role of testosterone. Front Neuroendocrinol. (2014) 35:42–57. doi: 10.1016/j.yfrne.2013.09.001 24076484 PMC3946856

[28] Altemus M Sarvaiya N Neill Epperson C. Sex differences in anxiety and depression clinical perspectives. Front Neuroendocrinol. (2014) 35:320–30. doi: 10.1016/j.yfrne.2014.05.004 PMC4890708 24887405

[29] Bresin K Schoenleber M. Gender differences in the prevalence of nonsuicidal self-injury: a meta-analysis. Clin Psychol Rev. (2015) 38:55–64. doi: 10.1016/j.cpr.2015.02.009 25795294

[30] Wan Y Chen R Wang S Clifford A Zhang S Orton S. Associations of coping styles with nonsuicidal self-injury in adolescents: do they vary with gender and adverse childhood experiences? Child Abuse Negl. (2020) 104:104470. doi: 10.1016/j.chiabu.2020.104470 32234639

[31] Garaigordobil M. Intrapersonal emotional intelligence during adolescence: sex differences, connection with other variables, and predictors. Eur J Investig Health Psychol Educ. (2020) 10:899–914. doi: 10.3390/ejihpe10030064 PMC8314288 34542518

[32] Lloyd E Kelley M Hope T. (1997). Self-mutilation in a community sample of adolescents: descriptive characteristics and provisional prevalence rates, in: Annual meeting of the Society for Behavioral Medicine, New Orleans, LA.

[33] Spitzer RL Kroenke K Williams JB Löwe B. A brief measure for assessing generalized anxiety disorder: the GAD-7. Arch Intern Med. (2006) 166:1092–7. doi: 10.1001/archinte.166.10.1092 16717171

[34] Plummer F Manea L Trepel D McMillan D. Screening for anxiety disorders with the GAD-7 and GAD-2: a systematic review and diagnostic metaanalysis. Gen Hosp Psychiatry. (2016) 39:24–31. doi: 10.1016/j.genhosppsych.2015.11.005 26719105

[35] Sousa TV Viveiros V Chai MV Vicente FL Jesus G Carnot MJ. Reliability and validity of the Portuguese version of the generalized anxiety disorder (GAD-7) scale. Health Qual Life Outcomes. (2015) 13:50. doi: 10.1186/s12955-015-0244-2 25908249 PMC4424548

[36] Burke TA Ammerman BA Hamilton JL Alloy LB. Impact of non-suicidal self-injury scale: initial psychometric validation. Cognit Ther Res. (2017) 41:130–42. doi: 10.1007/s10608-016-9806-9 PMC5560622 28824214

[37] Cohen J. Statistical power analysis for the behavioral sciences. London, England: Routledge (1988).

[38] Kim M Yu J. Factors contributing to non-suicidal self injury in Korean adolescents. J Korean Acad Community Health Nurs. (2017) 28:271–9. doi: 10.12799/jkachn.2017.28.3.271

[39] Marshall SK Tilton-Weaver LC Stattin H. Non-suicidal self-injury and depressive symptoms during middle adolescence: a longitudinal analysis. J Youth Adolesc. (2013) 42:1234–42. doi: 10.1007/s10964-013-9919-3 23371004

[40] Nixon MK Levesque C Preyde M Vanderkooy J Cloutier PF. The Ottawa Self-Injury Inventory: evaluation of an assessment measure of nonsuicidal self-injury in an inpatient sample of adolescents. Child Adolesc Psychiatry Ment Health. (2015) 9:26. doi: 10.1186/s13034-015-0056-5 26157482 PMC4495629

[41] McMahon EM Corcoran P McAuliffe C Keeley H Perry IJ Arensman E. Mediating effects of coping style on associations between mental health factors and self-harm among adolescents. Crisis. (2013) 34:242–50. doi: 10.1027/0227-5910/a000188 23357219

[42] Xiong W Liu H Gong P Wang Q Ren Z He M. Relationships of coping styles and sleep quality with anxiety symptoms among Chinese adolescents: a cross-sectional study. J Affect Disord. (2019) 257:108–15. doi: 10.1016/j.jad.2019.07.032 31301610

[43] Liu X Liu ZZ Fan F Jia CX. Menarche and menstrual problems are associated with non-suicidal self-injury in adolescent girls. Arch Womens Ment Health. (2018) 21:649–56. doi: 10.1007/s00737-018-0861-y 29804155

[44] Bronfenbrenner U. The Ecology of Human Development. Cambridge: Harvard University Press (1979). doi: 10.4159/9780674028845

[45] Ashiabi GS O’Neal KK. Child social development in context: an examination of some propositions in Bronfenbrenner’s bioecological theory. SAGE Open. (2015) 5:1–14. doi: 10.1177/2158244015590840

[46] Liu L Gu H Zhao X Wang Y. What contributes to the development and maintenance of school refusal in Chinese adolescents: a qualitative study. Front Psychiatry. (2021) 12:782605. doi: 10.3389/fpsyt.2021.782605 34975580 PMC8714792

[47] Liu L Wu J Wang J Wang Y Tong Y Ge C. What do Chinese families with depressed adolescents find helpful in family therapy? A Qualitative Study. Front Psychol. (2020) 11:1318. doi: 10.3389/fpsyg.2020.01318 32714235 PMC7344142

[48] Mink AJ Maddox MM Pinero AJZ Crockett EE. Gender differences in the physiological effects of emotional regulation. J Soc Psychol. (2023) 163:256–68. doi: 10.1080/00224545.2022.2064732 35527649

[49] Liu L Liu C Zhao X. Mapping the paths from styles of anger experience and expression to obsessive-compulsive symptoms: the moderating roles of family cohesion and adaptability. Front Psychol. (2017) 8:671. doi: 10.3389/fpsyg.2017.00671 28512441 PMC5411817

[50] Wang Y Gu H Zhao X Liu L. Chinese clients’ experiences throughout family therapy for school-refusing adolescents: A multiperspectival interpretative phenomenological analysis. Acta Psychologica. (2024) 243:104161. doi: 10.1016/j.actpsy.2024.104161 38280349

[51] Compas BE Jaser SS Bettis AH Watson KH Gruhn MA Dunbar JP. Coping, emotion regulation, and psychopathology in childhood and adolescence: a meta-analysis and narrative review. Psychol Bull. (2017) 143:939–91. doi: 10.1037/bul0000110 PMC7310319 28616996

[52] Aldinger M Stopsack M Barnow S Rambau S Spitzer C Schnell K. The association between depressive symptoms and emotion recognition is moderated by emotion regulation. Psychiatry Res. (2013) 205:59–66. doi: 10.1016/j.psychres.2012.08.032 22985543

